# Silver ion-mediated killing of a food pathogen: Melting curve analysis data of silver resistance genes and growth curve data

**DOI:** 10.1016/j.dib.2017.01.002

**Published:** 2017-01-11

**Authors:** Kuppan Gokulan, Katherine Williams, Sangeeta Khare

**Affiliations:** Division of Microbiology National Center for Toxicological Research, US Food and Drug Administration, Jefferson, AR 72079, USA

**Keywords:** Silver ion, Bacteria, Resistance, Melting curve, MIC

## Abstract

Limited antibacterial activity of silver ions leached from silver-impregnated food contact materials could be due to: 1) the presence of silver resistance genes *in tested bacteria*; or 2) lack of susceptibility to silver ion-mediated killing in the *bacterial* strain (K. Williams, L. Valencia, K. Gokulan, R. Trbojevich, S. Khare, 2016 [1]). This study contains data to address the specificity of silver resistance genes in *Salmonella* Typhimurium during the real time PCR using melting curve analysis and an assessment of the minimum inhibitory concentration of silver ions for *Salmonella*.

**Specifications Table**

**Table:** 

Subject area	*Biology*
More specific subject area	*Microbiology*
Type of data	*Images from Real-Time PCR and Graph*
How data was acquired	*Silver resistance gene data was acquired by conducting real time PCR experiment in ABI7500 machine and the raw images from ABI7500 were acquired. Data for* minimum inhibitory concentration (MIC) of silver ion for Salmonella Typhimurium *was assessed in Cytation (Biotek) and Graph was generated by Gen5 Microplate Reader and Imager Software from Biotek*
Data format	*Images (raw); Graph (analyzed)*
Experimental factors	*Silver resistance genes, bacterial growth*
Experimental features	*Assessment of silver resistance gene and susceptibility to silver ions in bacteria*
Data source location	*Jefferson, AR*
Data accessibility	*Data provided (as graph) and accessible within article*

**Value of the data**

• Presence of silver resistance gene(s) in a bacterial strain may cause resistance to silver ions or silver nanoparticle-mediated killing [2]
• Real time PCR-based data analysis is usually interpreted by threshold cycle; however these data can be unreliable in the absence of a melt curve analysis. Analysis of data generated by a melting curve of the silver resistance gene PCR end product provides specificity information for the amplicon.
• To assess the bactericidal effect of silver ions or silver nanoparticles, the test bacteria should be susceptible to silver ion-mediated killing.
• Data provided in this article showed that the minimum inhibitory concentration (MIC) of silver ion for *Salmonella* Typhimurium is similar to that published earlier for *E. coli* [3].

## 1 Data

**Fig. 1:**
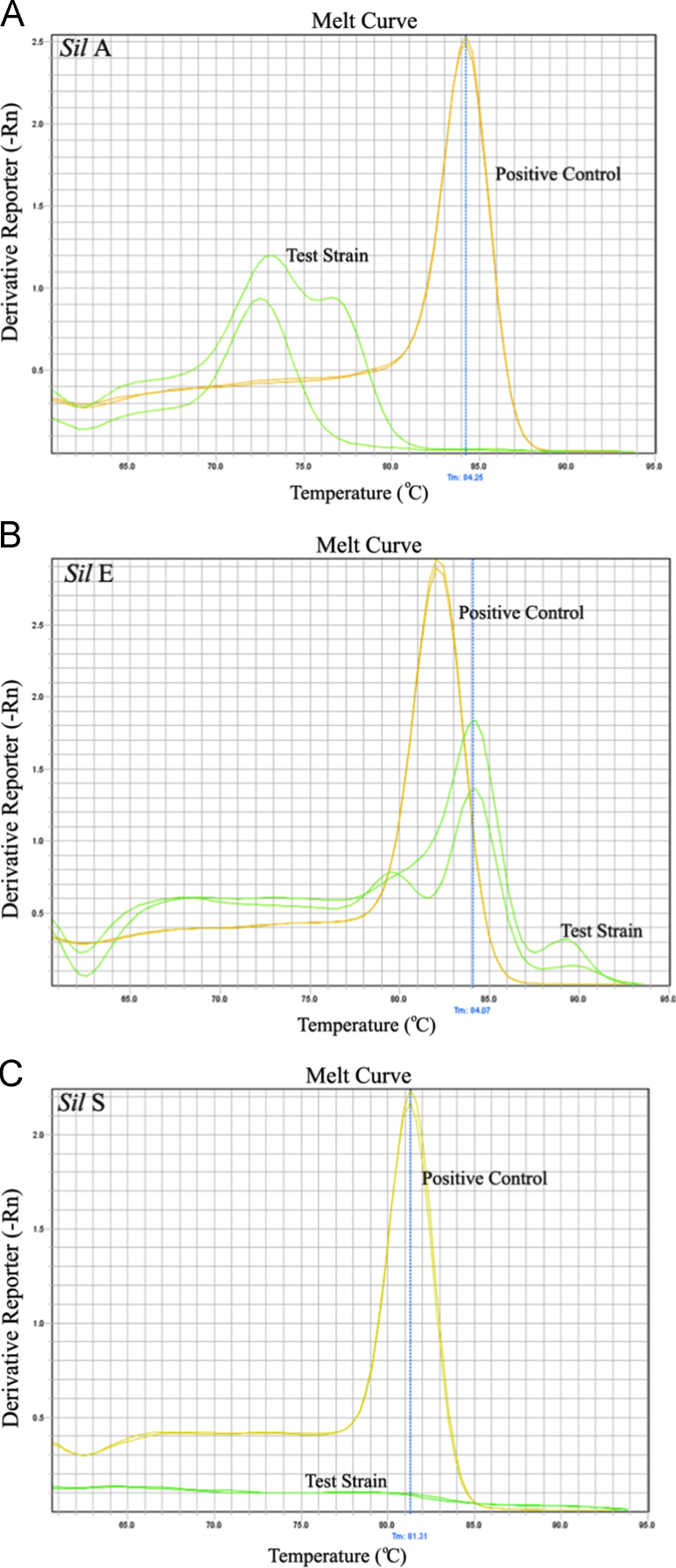
Melting curve analysis to determine the specificity of the PCR product. Sharp peak of the melting curve in the positive control confirms the specificity of the PCR product (mustard color peak in Figs. 1a, b, and c). However, a very different melting curve pattern was generated by the test strain for *sil*A and *sil*E (green color peaks in Figs. 1a and b). The melting curve was totally absent (green color line in Fig. 1c) for *sil*S gene in the test strain.(For interpretation of the references to color in this figure legend, the reader is referred to the web version of this article).

Silver resistance genes are part of a plasmid-associated gene cluster that encodes a silver binding protein (*silE*), efflux pump (*silA* and *silP*), and a membrane sensor kinase (*silS*) [2]. Melting curve analysis was performed to determine the specificity of the PCR products for the silver resistance genes (*silA, silE and silS*). The melting curve for the test strain was very different than the positive control strain, thus indicating that the test strain was negative for the presence of the silver resistance genes (Fig. 1).

**Fig. 2:**
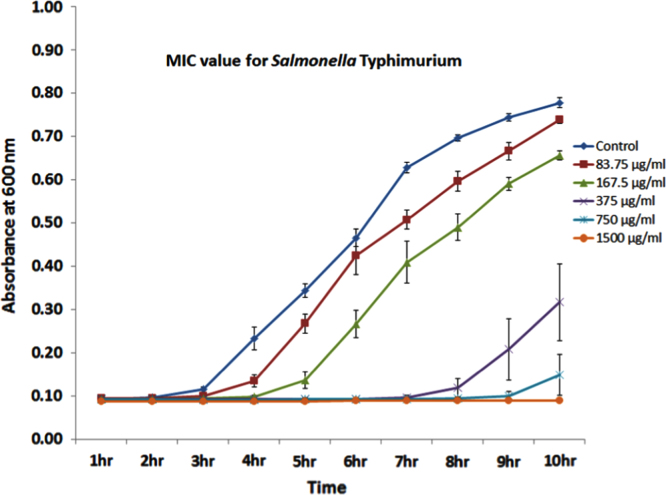
Minimum inhibitory concentration of silver ions for *Salmonella*. Several concentrations of silver nitrate were used to generate data for the MIC. Data is average of four replicates.

To address if the *Salmonella* strain was susceptible to silver ions, a growth curve analysis was performed to assess the MIC of silver ions. Several concentrations of silver nitrate (0.62 μg/ml to 1500 μg/ml) were incubated with the *Salmonella* Typhimurium at 37 °C and the growth of the bacteria was monitored for 10 h. Growth curve data showed that the silver ions at a concentration of 375 μg/ml inhibited/delayed the bacterial growth (Fig. 2). This concentration is similar to the silver ion concentration shown to be required to inhibit the growth of *E. coli* [3].

## 2 Experimental design, materials and methods

### 2.1 Melting curve analysis to determine the specificity of the PCR product

Real time PCR was used to confirm presence/absence of *silA, silE and silS* genes in test strain, as well as, in positive [*E. coli* (J53) with a plasmid (pMG101)] and negative (J53) controls (Williams et al., [1]). The Threshold Cycle for the negative strains and test strain were higher than positive control. This article provides the data to show the specificity of the PCR product. The melting curve was generated by a slow denaturing of the amplicon (ramp from 60 °C to 95 °C). A sharp peak in the fluorescence further confirms the specificity of amplicon.

### 2.2 Minimum inhibitory concentration of silver ions for Salmonella

Bacteria were grown overnight in Luria-Bertani (LB) broth at 37 °C and subcultured further to obtain the growth in the mid log phase. Ninety six well tissue culture plates were inoculated with a concentration of 4×10^4^ bacteria/well. Silver nitrate solution was used in a concentration range from (0.62 μg/ml to 1500 μg/ml). The plate was incubated at 37 °C in a Cytation instrument (from Biotek, Winooski, VT). Plate was read every 60 min for 10 h at 600 nm. Plates were shaken when not reading (continuous setting) at slow speed. Fig. 2 represented the growth curve data generated by incubation of silver nitrate above 83.74 μg/ml; below this concentration the growth curve pattern were similar to control. The data revealed that the minimum inhibitory concentration of silver ions for the bacteria was 375 μg/ml (Fig. 2).

## Disclaimer

The findings and opinions presented here represent the views of the authors. They do not necessarily reflect the views of the U.S. Food and Drug Administration.
